# Bone Regeneration Drug BMP-7 Mitigates Ponatinib-Induced Cardiotoxicity via Inhibition of Pyroptosis and Modulation of TGF-β/SMAD Signaling Pathway

**DOI:** 10.3390/cells15090762

**Published:** 2026-04-24

**Authors:** Jonatas M. Rolando, Dinender K. Singla

**Affiliations:** Division of Metabolic and Cardiovascular Sciences, Burnett School of Biomedical Sciences, College of Medicine, University of Central Florida, Orlando, FL 32816, USA; jonatas.demendoncarolando@ucf.edu

**Keywords:** cardiac inflammation, cardio-oncology, cell death, macrophage polarization, tyrosine kinase inhibitor

## Abstract

**Highlights:**

**What are the main findings?**

- Ponatinib induces cardiotoxicity via inflammation-driven pyroptosis.
- BMP-7 effectively counteracts ponatinib-induced cardiotoxicity.

**What are the implications of the main findings?**

- Identifies the pyroptotic pathway as a potential target to reduce ponatinib-induced cardiotoxicity.
- Establishes BMP-7 as a promising cardioprotective strategy for the treatment of ponatinib-induced cardiotoxicity.

**Abstract:**

Background: Ponatinib (PON), an effective tyrosine kinase inhibitor for leukemias harboring the T315I mutation, is limited by severe cardiotoxicity, including myocardial infarction and heart failure. Here, we investigated the therapeutic potential of Bone Morphogenetic Protein-7 (BMP-7), an anti-inflammatory growth factor, in a murine model of PON-induced cardiotoxicity. Methods: C57BL/6J mice were distributed into experimental groups receiving PON (25 mg/kg cumulative dose) either alone or with BMP-7 (600 μg/kg cumulative dose), along with a corresponding control group. Cardiac analyses included molecular and histological assessments. Results: PON administration induced a marked increase in monocyte infiltration and M1 macrophage polarization. These inflammatory events led to the upregulation of the pyroptotic cascade, leading to activation of the TGF-β1/SMAD2/3 signaling axis. In contrast, BMP-7 significantly attenuated these pathological responses by suppressing inflammation-induced pyroptosis and the TGF-β1/SMAD2/3 signaling axis. Conclusions: These findings identify inflammation-induced pyroptosis as a central driver of the pathological changes in PON-induced cardiotoxicity. Notably, our work highlights BMP-7’s capacity to inhibit these disease-related alterations. Collectively, these results expand on the current knowledge of the mechanistic framework of PON-induced cardiotoxicity, while also emphasizing BMP-7 as a promising therapeutic candidate with potential translational relevance.

## 1. Introduction

Cancer therapy has advanced substantially with the development and widespread adoption of targeted treatments such as tyrosine kinase inhibitors (TKIs) [1]. Despite their clinical success, many TKIs are associated with significant cardiotoxic effects, which can limit their therapeutic utility [2]. Ponatinib (PON), a 3rd generation TKI drug, was designed to treat chronic myeloid leukemia (CML) and Philadelphia chromosome-positive acute lymphoblastic leukemia (Ph+ALL) patients who harbor the T315I gatekeeper mutation, which confers resistance to earlier-generation TKIs [3,4,5]. Collectively, these two hematologic malignancies account for approximately 20% of new leukemia diagnoses in the United States [6,7]. PON was approved by the Food and Drug Administration (FDA) in 2012 and remains the treatment of choice for CML and Ph+ALL patients carrying the T315I gatekeeper mutation [8]. However, in 2013, reports of an increased incidence of severe vascular adverse events, including fatalities, prompted its temporary withdrawal from the market. Despite these safety concerns, the absence of comparably effective alternatives led to its reintroduction in early 2014 [9]. PON administration was related to markedly increased rates of fatal myocardial infarction, cardiomyopathy, and congestive heart failure, imposing substantial burdens on survivorship and quality of life during and after cancer treatment [10,11]. Recent evidence indicates that PON-induced cardiotoxicity involves several maladaptive cellular processes, including enhanced senescence, inflammation, and cellular stress [12,13,14,15]. However, despite these emerging insights, the underlying molecular processes driving cardiotoxicity induced by PON are not fully understood, being a major obstacle for the development of effective cardioprotective strategies.

Pyroptosis is an inflammation-driven cell death that is involved in cardiac homeostasis; however, its excessive activation has been implicated in pathological processes, including cardiac inflammation, remodeling, and dysfunction [16,17]. Canonical pyroptosis is defined by inflammasome assembly, followed by the downstream activation of caspase-1, Gasdermin D (GSDMD), interleukin-1 beta (IL-1β), and IL-18 [18]. GSDMD, the pyroptosis executor, undergoes caspase-1-mediated cleavage, allowing its N-terminal fragment to assemble into oligomeric complexes that disrupt membrane integrity, resulting in cell death and enhanced inflammatory signaling [19]. Nevertheless, it remains unclear whether the heightened inflammatory state observed in PON-induced cardiotoxicity can trigger pyroptotic cell death in the heart, and our study addresses this critical gap.

As previously mentioned, PON remains the most effective option for patients carrying the T315I mutation [20], underscoring the urgent need for alternative therapies capable of mitigating PON-induced cardiotoxicity. Recent investigations demonstrated the promising cardioprotective effects of different recombinant proteins in ameliorating therapy-induced cardiotoxicity [21,22]. Bone Morphogenetic Protein 7 (BMP-7) has received FDA approval for specific orthopedic indications, including long bone nonunion and posterolateral lumbar fusions [23,24]. Our previous work showed that BMP-7 can attenuate PON-induced cardiotoxicity by suppressing apoptosis and its associated signaling pathways [25]. However, it remains unknown whether BMP-7 can inhibit inflammation-associated pathophysiological changes in PON-induced cardiotoxicity.

In this study, we sought to (a) elucidate how PON orchestrates monocyte infiltration and shifts macrophages toward an M1 phenotype, culminating in inflammation-triggered pyroptotic death of cardiac cells; (b) elucidate the associated signaling mechanisms in PON-induced cardiotoxicity; (c) comprehend whether BMP-7 treatment can ameliorate PON-induced cardiotoxicity, through M2 macrophage polarization and inhibition of associated inflammatory signaling mechanisms.

## 2. Materials and Methods

### 2.1. Experimental Design

In this study, all animal procedures were performed under a protocol approved by the Institutional Animal Care and Use Committee (IACUC) at the University of Central Florida (UCF) and in accordance with the National Institutes of Health (NIH) guidelines. Animals were monitored regularly for any indications of illness or discomfort and euthanized humanely in accordance with the approved IACUC guidelines. In addition, the injection regimen employed in this study followed our previously published study [26]. Ponatinib was administered intraperitoneally to ensure rapid and consistent systemic exposure suitable for assessing its cardiotoxic effects. In contrast, BMP-7 was given intravenously to allow immediate circulation and efficient delivery to the cardiac tissue. The doses for PON and BMP-7 were selected in accordance with FDA-recommended guidelines for converting human equivalent doses to animal equivalent doses [27]. Moreover, the doses employed in this study fall within ranges previously evaluated in clinical trials [28,29].

All experimental animals were maintained under standardized housing conditions with controlled temperature and humidity, a 12 h light–dark cycle, and unrestricted access to food and water. All husbandry practices were kept consistent across groups to reduce variability. The number of animals per group was based on prior studies and standard experimental practices to provide adequate statistical power. In all experimental analyses, each group included seven mice, except for Western blotting, where each group included nine mice. Predefined inclusion and exclusion criteria were applied to determine which animals were eligible for analysis, with no mice excluded in this study.

**Figure 1:**
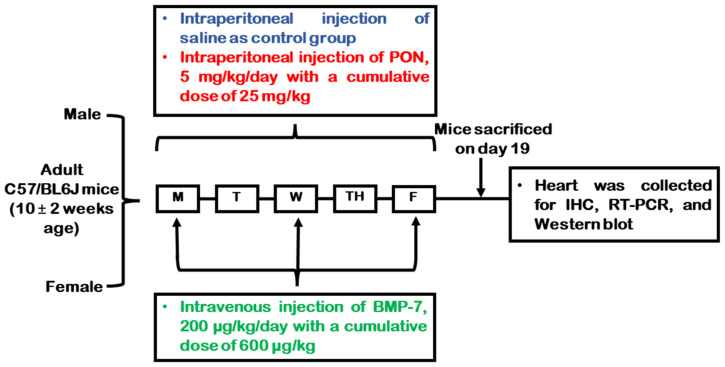
Study design schematic overview. Adult C57BL/6J mice received either saline, PON, or PON+BMP-7 according to the weekly dosing schedule. On D19, mice were euthanized, and hearts were collected for molecular and histological analyses.

In brief, wild-type C57BL/6J male and female mice weighing 20–25 g were randomly assigned into three experimental conditions (*n* = 9/group): a control group receiving normal saline, a PON-treated group, and a PON+BMP-7 group. Additionally, blinding was applied whenever possible to minimize experimental bias. Each animal served as its own experimental unit, and the number of animals (*n*) per group was reported for each analysis. Control and PON-treated mice received injections for five consecutive days. In the BMP-7 treatment group, mice were injected with PON for five days and with BMP-7 on three alternate days. Mice age, dosages, and injection details are shown in Figure 1.

On day 19 (D19), mice were euthanized under 4% isoflurane with subsequent cervical dislocation. After euthanasia, the hearts were removed, rinsed in 1× PBS to remove excess blood, and sectioned into two halves. The lower section was immersed in 4% paraformaldehyde (PFA) for fixation and histological processing, whereas the upper section was immediately frozen and stored at −80 °C for subsequent molecular assays. Outcome measures centered on assessing inflammatory responses and major molecular signaling pathways associated with ponatinib-induced cardiotoxicity and its attenuation by BMP-7. All chemicals used are listed in Table S1.

### 2.2. Tissue Processing

Fixed heart tissues were placed in a tissue processor for dehydration, clearing, and infiltration, and subsequently embedded in paraffin. Using a microtome, the paraffin-embedded heart tissues were cut into 5 μm sections and mounted onto microscope slides. All materials and equipment used are listed in Tables S1 and S2.

### 2.3. Immunohistochemistry (IHC) Staining

Double IHC staining was carried out following our previously described method [30]. Briefly, heart sections were incubated with cardiac sarcomeric-α-actin (Src-α-actin) using the M.O.M. (Mouse-on-Mouse) immunodetection kit reagents as recommended by the supplier. Then, sections were blocked with 10% normal goat serum (NGS), followed by incubation with primary antibodies, including: cluster of differentiation 14 (CD14), inducible nitric oxide synthase (iNOS), high mobility group box 1 (HMGB1), toll-like receptor 4 (TLR4), nucleotide-binding domain, leucine-rich-containing family, pyrin domain-containing-3 (NLRP3), caspase-1, IL-1β, IL-18, GSDMD, cluster of differentiation 206 (CD206), arginase-1, and IL-10. Then, a series of washes with 1× PBS was performed before incubation with the secondary antibody. Alexa Fluor 568 goat anti-rabbit antibody was used as a secondary antibody, followed by a series of washes with 1× PBS. Finally, the sections were mounted with 4′,6-diamidino-2-phenylindole (DAPI). A Keyence microscope was used for IHC quantification, with 20× images being analyzed through ImageJ (version 1.39o). Representative images were taken at 40×. The kit reagents and antibodies used for IHC staining are described in Tables S1 and S3.

### 2.4. Western Blotting

Western blotting was performed as previously described [31]. In short, 25 µg of protein were loaded onto Bis-Tris gels and run for 22 min. Following electrophoresis, proteins were transferred for 7 min. After that, the membranes were blocked with 5% bovine serum albumin (BSA) or 5% non-fat milk (NFM), followed by overnight primary antibody incubation, including CD14, iNOS, HMGB1, TLR4, NLRP3, caspase-1, IL-1β, IL-18, GSDMD, CD206, arginase-1, transforming growth factor-beta 1 (TGF-β1), phosphorylated suppressor of mothers against decapentaplegic 2 (pSMAD2), pSMAD3, and glyceraldehyde-3-phosphate dehydrogenase (GAPDH). Secondary antibody incubation was performed using an HRP-linked anti-rabbit IgG antibody, and the membranes were subsequently imaged. Membranes were stripped and reprobed to detect different proteins on the same blot. Band intensities were quantified using ImageJ software. GAPDH served as the internal loading control for normalization, and all values are reported as arbitrary units (A.U.). A list with all antibodies (Table S3) and equipment (Table S2) used is provided in Supplementary Materials.

### 2.5. Real-Time Polymerase Chain Reaction (RT-PCR)

Total RNA was extracted from cardiac tissues, followed by complementary DNA (cDNA) synthesis. Then, quantitative RT-PCR was carried out, following previously described procedures [30]. Gene expression was assessed using mouse-specific primer sets (Table S4), including tumor necrosis factor-alpha (TNF-α), IL-6, NLRP3, caspase-1, IL-1β, IL-18, GSDMD, IL-10, and GAPDH. After reaction, melt-curve analysis was performed to verify reaction specificity. Gene expression fold changes were calculated using the 2^−ΔΔCT^ method, with GAPDH serving as the normalizing control. This housekeeping gene ensures that differences in mRNA levels reflect experimental treatments rather than variations in sample loading. All chemicals and instruments used are described in Tables S1 and S2.

### 2.6. Statistical Analysis

The differences among groups were assessed using a one-way ANOVA with Tukey’s multiple-comparison test, implemented in GraphPad Prism (version 10.1.0). A *p*-value below 0.05 was considered statistically significant. Values were expressed as mean ± standard error of the mean (SEM).

## 3. Results

### 3.1. BMP-7 Treatment Reduces PON-Induced Monocyte Infiltration and M1 Macrophage Polarization

**Figure 2:**
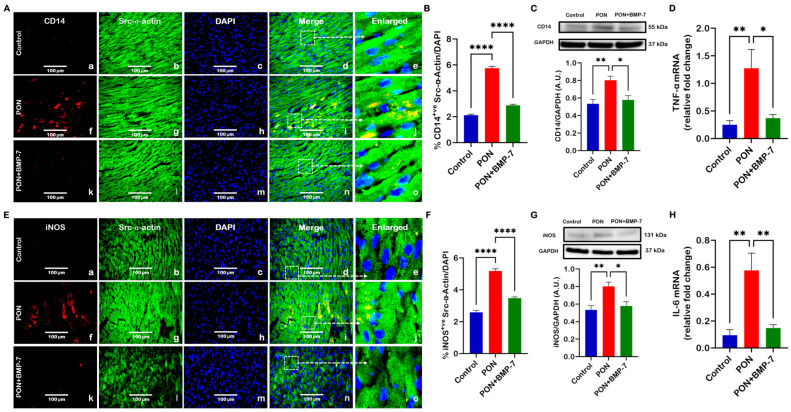
BMP-7 treatment reduces PON-induced monocyte infiltration and M1 macrophage polarization. PON increases monocyte infiltration (CD14, (**A**)) and M1 macrophage polarization (iNOS, (**E**)), which are reduced by BMP-7. CD14 and iNOS are shown in red (a, f, k). Cardiomyocytes are labeled in green with Src-α-actin (b, g, l), and nuclei are stained blue with DAPI (c, h, m). Merged images (d, i, n) and corresponding magnified views (e, j, o) highlight the areas marked by dashed boxes. Scale bar = 100 μm. (**B**,**F**) IHC quantification (*n* = 7/group). (**C**,**G**) Representative blots and densitometry (*n* = 6–7/group, A.U.). (**D**,**H**) TNF-α and IL-6 gene expression (*n* = 5–7/group). Results are shown as mean ± SEM. * *p* < 0.05, ** *p* < 0.01, and **** *p* < 0.0001.

Our earlier findings demonstrated that PON triggers apoptosis in cardiac cells [25], a mechanism that promotes cardiac inflammation [32]. Here, we aimed to investigate whether PON induces an inflammatory response, enhances monocyte infiltration, and inflammatory macrophage polarization in the heart. IHC photomicrographs revealed an increase in CD14 (Figure 2A), a marker for monocytes, in PON-treated hearts (f–j) relative to control (a–e). Interestingly, we observed a decrease in CD14 within the BMP-7 treatment group (k–o) in comparison with the PON group, indicating reduced infiltration of monocytes in cardiac tissue. We also observed a similar trend in iNOS (Figure 2E), a marker for pro-inflammatory M1 macrophages, suggesting that PON can promote M1 polarization in the heart. Monocytes and M1 macrophages, stained in red, could be observed infiltrating cardiomyocytes, which were stained in green with Src-α-actin. Additionally, our IHC quantitative data confirmed significantly higher expression of CD14 (Figure 2B) and iNOS (Figure 2F) in PON as opposed to control, while expression levels were significantly lower in the PON+BMP-7 group. Further, we performed Western blotting, and our densitometric analysis displayed a similar trend with increased protein expression of CD14 (Figure 2C) and iNOS (Figure 2G) in the PON group, which was reduced by BMP-7 treatment.

Next, we evaluated the pro-inflammatory cytokines TNF-α and IL-6, which are key markers associated with M1 macrophages [33]. Compared with controls, the PON group revealed a significant upregulation in gene expression of TNF-α (Figure 2D) and IL-6 (Figure 2H). In contrast, BMP-7-treated hearts had decreased gene expression of both cytokines relative to the PON group. Collectively, the data indicate that PON can elicit monocyte infiltration and M1 polarization, while increasing pro-inflammatory cytokine secretion in cardiac tissue; notably, BMP-7 treatment mitigates these responses.

### 3.2. BMP-7 Treatment Inhibits PON-Induced HMGB1-TLR4 Signaling and Pyroptosis Initiation

**Figure 3:**
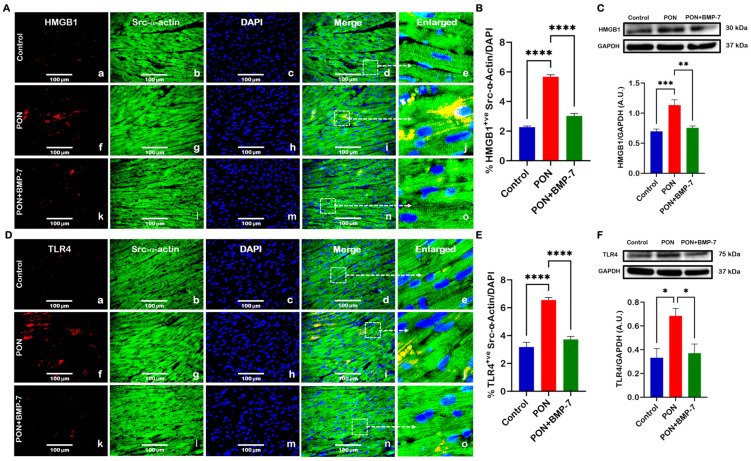
BMP-7 treatment inhibits PON-induced HMGB1-TLR4 signaling and pyroptosis initiation. (**A**,**D**) Representative photomicrographs of HMGB1 and TLR4 IHC staining. HMGB1 and TLR4 are shown in red (a, f, k). Cardiomyocytes are labeled in green with Src-α-actin (b, g, l), and nuclei are stained blue with DAPI (c, h, m). Merged images (d, i, n) and magnified panels (e, j, o) provide enhanced clarity and facilitate accurate identification of marker distribution within the cardiac tissue. Scale bar = 100 μm. (**B**,**E**) IHC quantitative analysis (*n* = 7/group). (**C**,**F**) Immunoblots and protein quantification of HMGB1 (*n* = 6–7/group, A.U.) and TLR4 (*n* = 6/group, A.U.). Results are shown as mean ± SEM. * *p* < 0.05, ** *p* < 0.01, *** *p* < 0.001, and **** *p* < 0.0001.

Next, given that sterile inflammation can initiate pyroptotic cell death [34], we sought to determine whether PON administration can initiate pyroptotic signaling in the heart. IHC images revealed an augmented expression of HMGB1 (Figure 3A) and TLR4 (Figure 3D) in PON-treated hearts (f–j) relative to control (a–e), which was additionally confirmed with quantification (Figure 3B,E). Contrarily, PON+BMP-7 group (k–o) cardiomyocytes showed reduced expression of HMGB1 and TLR4. Further, our Western blotting analysis confirmed higher protein levels of HMGB1 (Figure 3C) and TLR4 (Figure 3F) in PON-treated hearts in comparison with controls. Whereas decreased protein levels were observed with BMP-7 treatment. Our findings indicate that PON activates upstream signaling of the pyroptotic pathway, whereas BMP-7 effectively inhibits this signaling.

### 3.3. BMP-7 Treatment Ameliorates PON-Induced Pyroptotic Cell Death

**Figure 4:**
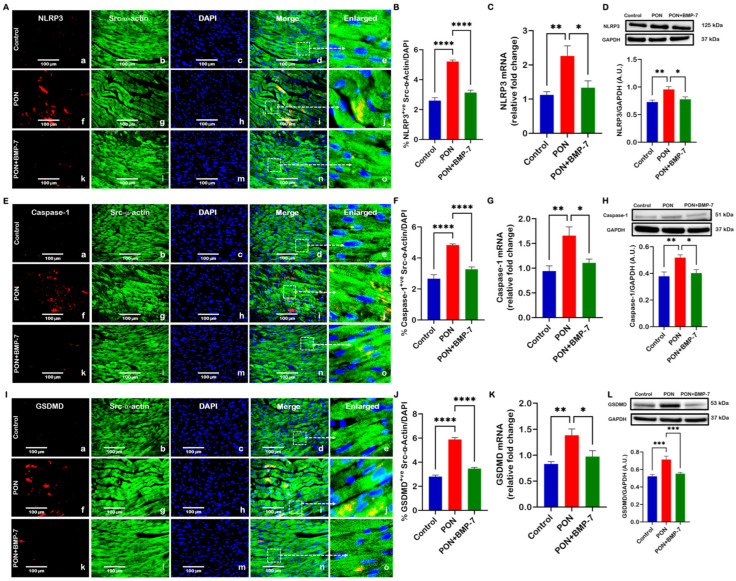
BMP-7 treatment ameliorates PON-induced pyroptotic cell death. (**A**,**E**,**I**) PON increases NLRP3, caspase-1, and GSDMD expression, which is reduced by BMP-7. NLRP3, caspase-1, and GSDMD are shown in red (a, f, k). Cardiomyocytes are labeled in green with Src-α-actin (b, g, l), and nuclei are stained blue with DAPI (c, h, m). Merged images (d, i, n) and corresponding magnified views (e, j, o) highlight the areas marked by dashed boxes. Scale bar = 100 μm. (**B**,**F**,**J**) IHC staining quantification (*n* = 7/group). (**C**,**G**,**K**) Relative mRNA expression (*n* = 5–6/group). (**D**,**H**,**L**) Western blots and densitometric analysis (*n* = 6–8/group, A.U.). Results are shown as mean ± SEM. * *p* < 0.05, ** *p* < 0.01, *** *p* < 0.001, and **** *p* < 0.0001.

Given that NLRP3 inflammasome formation is a critical event in pyroptosis [35], we assessed whether PON-induced pyroptosis initiation stimulates NLRP3 formation and downstream pyroptosis processes in the heart. Representative images showed higher expression of NLRP3^+ve^ (Figure 4A), caspase-1^+ve^ (Figure 4E), and GSDMD^+ve^ (Figure 4I) in the PON group (f–j), as opposed to control (a–e). Interestingly, BMP-7 treatment (k–o) was accompanied by diminished expression of these markers. Additionally, the PON group revealed increased levels of all markers (Figure 4B,F,J) within its cardiomyocytes in our IHC quantification, whereas the PON+BMP-7 group showed reduced expression.

Furthermore, we performed RT-PCR (Figure 4C,G,K) and observed upregulation in gene expression of NLRP3, caspase-1, and GSDMD in the PON group as opposed to controls, which were downregulated by BMP-7 treatment. Our Western blotting analysis (Figure 4D,H,L) further confirmed that the protein expression for all markers increased in PON-treated hearts, whereas it was reduced with BMP-7 treatment. Collectively, these results indicate that PON’s cardiotoxic effects can arise from NLRP3 inflammasome formation and the pyroptosis executor GSDMD activation, whereas BMP-7 treatment effectively inhibits this process.

### 3.4. BMP-7 Attenuates PON-Induced Expression of Pyroptosis-Associated Cytokines

**Figure 5:**
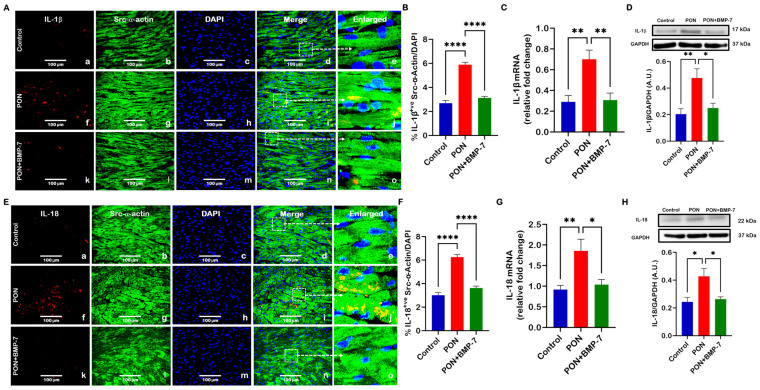
BMP-7 attenuates PON-induced expression of pyroptosis-associated cytokines. (**A**,**E**) IHC staining of IL-1β and IL-18 in cardiac sections. IL-1β and IL-18 are shown in red (a, f, k). Cardiomyocytes are labeled in green with Src-α-actin (b, g, l), and nuclei are stained blue with DAPI (c, h, m). Merged images (d, i, n) and enlarged images (e, j, o) enable precise localization and clearer visualization of the marker expression within the cardiac tissue. Scale bar = 100 μm. (**B**,**F**) IHC quantification (*n* = 7/group). (**C**,**G**) Relative gene expression levels of IL-1β (*n* = 6–7/group) and IL-18 (*n* = 5–6/group). (**D**,**H**) Representative blots and protein quantification of IL-1β (*n* = 6–7/group, A.U.) and IL-18 (*n* = 6/group, A.U.). Results are shown as mean ± SEM. * *p* < 0.05, ** *p* < 0.01, and **** *p* < 0.0001.

The N-terminal fragment produced following GSDMD cleavage forms membrane-associated oligomers that generate pores, enabling the release of pro-inflammatory cytokines [36]. To determine whether PON promotes pyroptosis-associated cytokines in the heart, we evaluated the expressions of IL-1β and IL-18. Our IHC images showed that the PON group (f–j) had increased expression of IL-1β (Figure 5A) and IL-18-positive cardiomyocytes (Figure 5E) in comparison to the control (a–e), which was confirmed in quantitative analysis (Figure 5B,F). In contrast, BMP-7 treatment (k–o) reduced the number of positive cardiomyocytes for both cytokines.

Consistent with these findings, RT-PCR analysis (Figure 5C,G) demonstrated upregulation of IL-1β and IL-18 mRNA levels in the PON group in comparison to controls, which were significantly downregulated upon BMP-7 treatment. Western blot analysis (Figure 5D,H) further confirmed increased protein expression of IL-1β and IL-18 in PON-treated hearts, which was markedly diminished with BMP-7 treatment. The observed outcomes highlight that PON-induced cardiotoxicity can be associated with activation of pyroptotic cell death and increased expression of pyroptotic inflammatory cytokines, while BMP-7 effectively suppresses this pyroptosis-mediated inflammatory response.

### 3.5. BMP-7 Treatment Enhances M2 Macrophage Polarization

**Figure 6:**
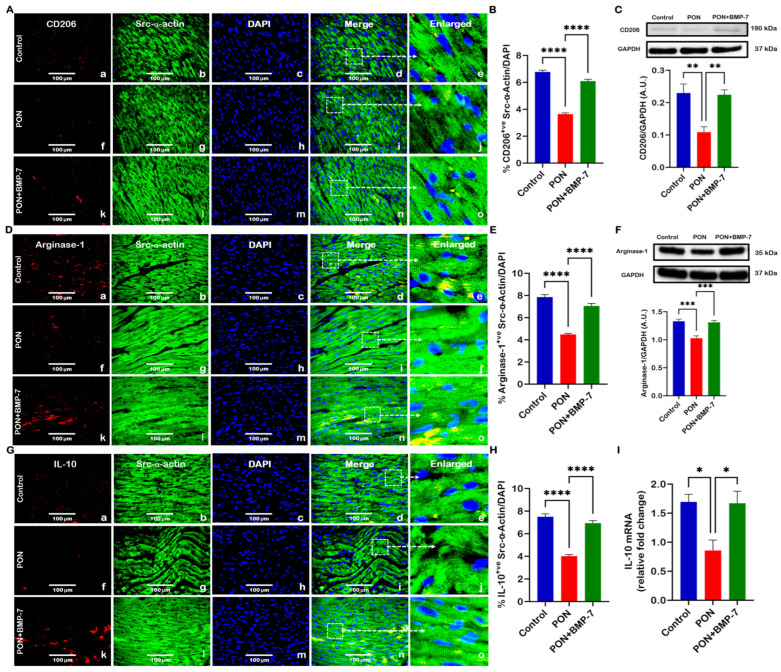
BMP-7 treatment enhances M2 macrophage polarization. (**A**,**D**,**G**) Representative immunofluorescence images showing CD206, arginase-1, and IL-10 stain. Red fluorescence indicates CD206, arginase-1, and IL-10 expression (a, f, k). Cardiomyocytes are visualized in green using Src-α-actin labeling (b, g, l), while nuclei are counterstained blue with DAPI (c, h, m). Merged images are shown in panels (d, i, n), and enlarged views (e, j, o) provide detail of the regions outlined by dashed boxes. Scale bar = 100 μm. (**B**,**E**,**H**) IHC quantitative analysis (*n* = 7/group). (**C**,**F**) Western blots and densitometric analysis for CD206 and arginase-1 (*n* = 6–7/group, A.U.). (**I**) Relative IL-10 mRNA expression (*n* = 5/group). Results are shown as mean ± SEM. * *p* < 0.05, ** *p* < 0.01, *** *p* < 0.001, and **** *p* < 0.0001.

Several reports indicate that BMP-7 drives macrophages toward an M2 anti-inflammatory state [37,38]. Accordingly, we sought to determine whether BMP-7 can promote M2 macrophage polarization in PON-induced cardiotoxicity. Microscopic images showed lower expression of CD206 (M2 macrophage marker, Figure 6A) and arginase-1 (M2 macrophage marker, Figure 6D) in PON-treated hearts (f–j), in comparison to control (a–e). In contrast, an enhanced expression was detected in BMP-7-treated hearts (k–o). When compared to controls, the PON group revealed a decrease in CD206 (Figure 6B) and arginase-1 (Figure 6E) expression in our IHC quantification, which was increased with BMP-7 treatment. M2 macrophages, stained in red, could be observed between cardiomyocytes, which were stained in green with Src-α-actin. Further, there was a significant decline in the protein expression of CD206 (Figure 6C) and arginase-1 (Figure 6F) in the PON group heart tissues, which was increased with BMP-7 treatment.

We subsequently assessed IL-10, an anti-inflammatory cytokine linked to the M2 macrophage phenotype [39]. Our microscopic fluorescence images revealed lower expression of IL-10 (Figure 6G) in PON-administered animals, which was increased in the PON+BMP-7 group and confirmed in quantification (Figure 6H). Additionally, RT-PCR analysis also showed downregulation of IL-10 (Figure 6I) in the PON group, whereas the PON+BMP-7 group revealed upregulation of IL-10. Thus, our data indicate that BMP-7 treatment can promote M2 polarization in the cardiac tissue of PON-administered mice, thereby potentially dampening PON-mediated inflammatory signaling.

### 3.6. BMP-7 Treatment Inhibits PON-Activated TGF-β1/SMAD2/3 Signaling Pathway

**Figure 7:**
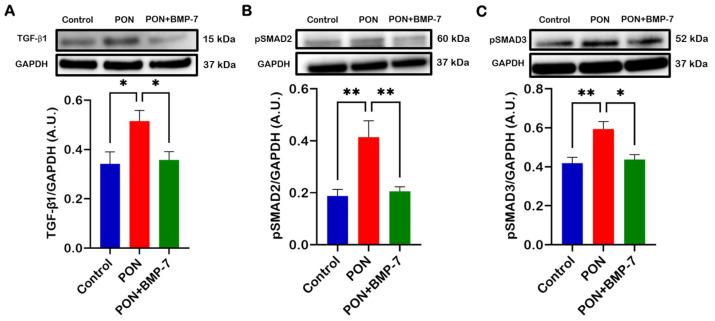
BMP-7 treatment inhibits PON-activated TGF-β1/SMAD2/3 signaling pathway. (**A**–**C**) Immunoblots and protein quantitative analysis of TGF-β1 (*n* = 6/group), pSMAD2 (*n* = 6/group), and pSMAD3 (*n* = 6–7/group). Data are expressed as A.U. and shown as mean ± SEM. * *p* < 0.05 and ** *p* < 0.01.

To investigate the role of the TGF-β/SMAD signaling pathway in PON-induced cardiotoxicity, we performed Western blotting. Our analysis showed that PON administration resulted in upregulation of TGF-β1, pSMAD2, and pSMAD3 proteins (Figure 7A–C), while BMP-7 treatment suppressed this increase. Taken together, our results indicate that PON can exert its cardiotoxic effects via the TGF-β1/SMAD2/3 signaling cascade, whereas BMP-7 treatment inhibits this pathway.

## 4. Discussion

PON was developed to overcome the T315I-mediated resistance in CML and Ph+ALL [40,41]. During clinical trials, PON was associated with different cardiac side effects such as hypertension, arrhythmia, myocardial infarction, and heart failure, which hinder its use for leukemia treatment [42]. However, limited studies elucidated PON-induced cardiotoxicity mechanisms [12,13,14,15,41]. Therefore, in this study, we (a) showed that PON is a key regulator of inflammatory macrophage polarization and a critical contributor to cardiac inflammation, (b) established PON-induced pyroptosis and associated TGF-β1/SMAD2/3 signaling pathway, and (c) showed that BMP-7 treatment can counteract PON side effects, leading to anti-inflammatory macrophage polarization.

Our previous work demonstrated that systemic PON administration is associated with increased apoptotic cell death in cardiac tissue [25]. This process is known to trigger monocyte recruitment and amplify cardiac inflammation during heart failure [43,44,45]. However, it is still unknown whether PON administration can drive these events in the hearts of C57BL/6J mice. In this way, our work establishes an upregulation of monocyte infiltration and M1 macrophage polarization alongside upregulation of TNF-α and IL-6 in PON-treated hearts. Supporting these observations, a recent study has shown that apolipoprotein E (ApoE) KO mice treated with PON had increased cardiac levels of pro-inflammatory cytokines and M1 macrophages [13]. Together, these findings expand on the pro-inflammatory consequences of PON administration. Further, we established the consequences of increased inflammation on cardiomyocytes.

Pyroptosis arises as an inflammation-driven cell death that can be activated by damage-associated molecular patterns (DAMPs), such as HMGB1, binding to pattern recognition receptors (PRRs) like TLR4 [46]. Activation of TLR4 triggers downstream NLRP3 inflammasome assembly, which promotes caspase-1 formation and subsequently cleavage of GSDMD. Simultaneously, caspase-1 promotes the maturation of IL-1β and IL-18, intensifying the inflammatory cascade [47]. Nevertheless, it remains to be determined whether cardiac inflammation induced by PON can trigger pyroptotic cell death in the heart. Our study addresses this question and demonstrates that PON-treated hearts showed increased HMGB1-TLR4 signaling, along with upregulation of the NLRP3 inflammasome and pyroptosis executor GSDMD, indicating robust activation of the pyroptotic machinery. These findings align with a previous report showing that ApoE KO mice treated with PON exhibit elevated cardiac expression of TLR4, NLRP3, and IL-1β [13], reinforcing that PON promotes inflammasome activation as a key driver of cardiac inflammation and injury. Collectively, these results offer initial direct evidence that PON-induced cardiotoxicity is mediated, at least in part, by heightened inflammatory responses leading to pyroptotic cell death.

TGF-β1, the predominant isoform expressed in cardiac tissue, activates its downstream signaling cascade by phosphorylating SMAD2 and SMAD3. These phosphorylated SMADs then bind to SMAD4 and translocate to the nucleus to promote the expression of extracellular matrix proteins such as fibronectin and collagen, while also modulating cytokine production and release [48]. Nonetheless, it remains unknown whether PON-induced cardiotoxicity is mediated through the TGF-β1/SMAD2/3 signaling pathway. Our study is the first to demonstrate marked increases in TGF-β1, pSMAD2, and pSMAD3 protein expression under PON administration. These results align with observations from a dasatinib study, a 2nd generation TKI, showing upregulation of TGF-β1 and fibronectin in human vascular endothelial cells [49]. Taken together, our study reveals that activation of the TGF-β1/SMAD2/3 signaling cascade may underlie the cardiotoxicity associated with PON administration.

TGF-β1 upregulation was observed in different animal models of cardiac remodeling and patients with hypertrophic cardiomyopathy [50]. In line with this, our previous work demonstrated that PON administration contributes to the progression of adverse cardiac remodeling [25]. Specifically, we showed that PON treatment was associated with increased heart weight ratio and the development of cardiac hypertrophy and fibrosis; importantly, these structural changes were accompanied by significant impairments in cardiac function, consistent with previous reports [13,51,52]. Collectively, these data indicate that PON-induced cardiac inflammation can disrupt cardiac homeostasis, leading to maladaptive remodeling and ultimately progressing to functional cardiac dysfunction.

As previously discussed, PON-associated side effects remain a significant clinical challenge, and there are no FDA-approved therapies to prevent or mitigate PON-induced cardiotoxicity. Given this unmet need, we evaluated the therapeutic promise of BMP-7, a naturally occurring growth factor that has been evaluated in multiple clinical trials and demonstrated an excellent safety profile with no reported toxicity [53,54]. BMP-7 exerts its biological effects in cardiomyocytes by binding to its receptor, BMP Receptor Type 2 (BMPR2), which oligomerizes with BMPR1 to phosphorylate SMAD1, SMAD5, and SMAD8. These phosphorylated SMADs form the SMAD1/5/8 complex, which associates with SMAD4 and translocates to the nucleus, where it induces mitogenic, anti-inflammatory, and anti-fibrotic gene expression, suppressing the TGF-β1-SMAD2/3 signaling pathway [55,56,57]. Beyond the cardiovascular system, BMP-7 therapy has demonstrated beneficial effects across a broad spectrum of pathological conditions, including diabetic wound healing, Graves’ orbitopathy, pulmonary fibrosis, Parkinson’s disease, tibial non-unions, and myocardial fibrosis, highlighting its pleiotropic therapeutic potential [37,58,59,60,61,62].

M2 macrophages are distinguished by their secretion of IL-10, a key anti-inflammatory mediator, which prevents maladaptive tissue remodeling and dampens pro-inflammatory signaling [63,64]. However, the capacity of BMP-7 to promote M2 macrophage polarization under PON-induced cardiotoxicity has not yet been established. The present study provides the first evidence that BMP-7 treatment markedly upregulated M2 macrophage markers, including CD206 and arginase-1, under PON administration. Collectively, these findings indicate that BMP-7 reduces PON-induced inflammation while promoting macrophage polarization toward a cardioprotective phenotype.

In addition to its immunomodulatory effects, BMP-7 treatment attenuated key intracellular pathological processes associated with PON-induced cardiotoxicity. Our novel study demonstrated that BMP-7 significantly reduced the activation of pyroptotic cell death and its downstream effector markers, while suppressing the TGF-β1/SMAD2/3 signaling cascade. These observations are in line with established evidence showing that BMP-7 inhibits cardiac pyroptosis in a diabetic mouse model [65]. In addition, our previous work demonstrated that BMP-7 effectively reversed PON-induced maladaptive cardiac remodeling and rescued the associated cardiac dysfunction [25]. Taken together, our results highlight BMP-7 as a promising therapeutic candidate capable of counteracting multiple pathological mechanisms, thereby offering a comprehensive strategy for treating PON-induced cardiotoxicity.

Our findings highlight the therapeutic promise of BMP-7 in attenuating PON-associated cardiotoxic effects. However, the current study displays some limitations. First, it relies on preclinical murine models, highlighting the need for future loss- or gain-of-function experiments using human cardiomyocytes to strengthen translational applicability and establish a causal relationship. Future studies are also required to understand how BMP-7-driven immunomodulation and cardiomyocyte protection pathways intersect in PON-induced cardiotoxicity. Also, our study lacks a BMP-7-only group, although a previous study has shown an absence of dosage toxicity [54], and unpublished data from our lab show no significant differences in cardiac function between the BMP-7-only group and control animals. Another limitation is that this study did not perform sex-specific analyses, as male and female mice were evaluated together. Given the potential for sex-dependent variation in cardiac responses, future investigations should address this factor. Additionally, future studies with CML and Ph+ALL mouse models will be important to confirm that BMP-7 does not interfere with PON’s anti-cancer effects. Despite these constraints, our findings uncover new mechanistic insights into PON-induced cardiotoxicity and underscore the protective effects of BMP-7 within preclinical research.

## 5. Conclusions

In conclusion, our findings demonstrate for the first time that PON exerts its cardiotoxic effects by promoting inflammation-driven pyroptotic cell death. This was evidenced by enhanced inflammation in the heart, with upregulation of M1 macrophages, pro-inflammatory cytokines, and pyroptotic markers. Further, we unveiled the TGF-β/SMAD pathway as a key modulator of these cardiotoxic effects. In contrast, we observed that BMP-7 was able to counteract PON-induced cardiotoxicity through inhibition of inflammation and pyroptotic-associated pathways. In addition, BMP-7 treatment induced macrophage polarization towards an anti-inflammatory state. Collectively, our work provides novel insights into PON-induced cardiotoxicity mechanisms and emphasizes BMP-7 as a potential therapeutic option to mitigate these cardiotoxic effects, contributing to the advancement of safer and more effective clinical strategies.

## Data Availability

The data presented in this study are available on request from the corresponding author.

## Supplementary Materials

The following supporting information can be downloaded at: https://www.mdpi.com/article/10.3390/cells15090762/s1, Table S1: List of Chemicals, Materials, and Kits Used in the Study; Table S2: List of Machines Used in the Study; Table S3: List of Antibodies Used in the Study; Table S4: List of Primers Used in the Study.

## Abbreviations

The following abbreviations are used in this manuscript:

ANOVA	Analysis of variance
ApoE	Apolipoprotein E
AU	Arbitrary units
BMP-7	Bone Morphogenetic Protein 7
BMPR1	Bone Morphogenetic Protein Receptor Type 1
BMPR2	Bone Morphogenetic Protein Receptor Type 2
BSA	Bovine serum albumin
cDNA	Complementary DNA
CD14	Cluster of differentiation 14
CD206	Cluster of differentiation 206
CML	Chronic myeloid leukemia
D19	Day 19
DAMPs	Damage-associated molecular patterns
DAPI	4′,6-diamidino-2-phenylindole
FDA	Food and Drug Administration
GAPDH	Glyceraldehyde-3-phosphate dehydrogenase
GSDMD	Gasdermin D
HMGB1	High mobility group box 1
IACUC	Institutional Animal Care and Use Committee
IHC	Immunohistochemistry
IL-1β	Interleukin-1 beta
IL-6	Interleukin-6
IL-10	Interleukin-10
IL-18	Interleukin-18
iNOS	Inducible nitric oxide synthase
MOM	Mouse on Mouse
NCI	National Cancer Institute
NFM	Non-fat milk
NGS	Normal goat serum
NIDKK	National Institutes of Diabetes and Digestive and Kidney Diseases
NIH	National Institutes of Health
NLRP3	Nucleotide-binding domain, leucine-rich-containing family, pyrin domain-containing-3
PBS	Phosphate-buffered saline
PFA	Paraformaldehyde
Ph+ALL	Philadelphia-positive acute lymphoblastic leukemia
PON	Ponatinib
PRRs	Pattern recognition receptors
pSMAD2	Phosphorylated suppressor of mothers against decapentaplegic 2
pSMAD3	Phosphorylated suppressor of mothers against decapentaplegic 3
RT-PCR	Real-time polymerase chain reaction
SEM	Standard error of the mean
SMAD1	Suppressor of mothers against decapentaplegic 1
SMAD2	Suppressor of mothers against decapentaplegic 2
SMAD3	Suppressor of mothers against decapentaplegic 3
SMAD4	Suppressor of mothers against decapentaplegic 4
SMAD5	Suppressor of mothers against decapentaplegic 5
SMAD8	Suppressor of mothers against decapentaplegic 8
Src-α-actin	Sarcomeric-α-actin
TGF-β	Transforming growth factor
TGF-β1	Transforming growth factor beta 1
TKIs	Tyrosine kinase inhibitors
TLR4	Toll-like receptor 4
TNF-α	Tumor necrosis factor-alpha
UCF	University of Central Florida
