# Supplementary material for: Bone Regeneration Drug BMP-7 Mitigates Ponatinib-Induced Cardiotoxicity via Inhibition of Pyroptosis and Modulation of TGF-β/SMAD Signaling Pathway
Source: Cells. 2026 Apr 24;15(9):762. doi: 10.3390/cells15090762 (PMC13162625; doi:10.3390/cells15090762)
Supplement: Supplementary file 1 [file cells-15-00762-s001.zip › cells-4235317-supplementary.pdf]

Table S1. List of Chemicals, Materials, and Kits Used in the Study

Chemical and Kits	Supplier and Cat. No.
PON	Selleckchem; Cat. #S1490
BMP-7	Bioclone; Cat. #PA-0401
ColorFrost™ Plus	Thermo Fisher Scientific; #12-550-16A
M.O.M. (Mouse on Mouse) Immunodetection Kit	Vector Laboratories; Cat. #BMK-2202
Normal Goat Serum	Vector Laboratories; Cat. #S-1000-20
DAPI	Vector Laboratories; Cat. #H-1200
Bolt™ Bis-Tris Gels	Thermo Fisher Scientific; Cat. NW04120BOX
Restore™ Western Blot Stripping Buffer	Thermo Fisher Scientific; Cat. 21063
Pierce™ ECL Western Blotting Substrate	Thermo Fisher Scientific; Cat. 32106
TRIzol™ Reagent	Invitrogen; Cat. 15-596-018
SuperScript™ III First-Strand Synthesis SuperMix	Invitrogen; Cat. 18080400
SYBR GreenER™ qPCR SuperMix Universal	Invitrogen; Cat. 11762500
Hematoxylin	Epredia; Cat. #7211
Acid Alcohol	Poly Scientific R&D Corp; Cat. #S104
Bluing Reagent	Thermo Fisher Scientific; Cat. #7301
Eosin	Epredia; Cat. #6766008
Bouin's Fixative	Poly Scientific R&D Corp; Cat. #S129
Weigert's Iron Hematoxylin Solutions A&B	Poly Scientific R&D Corp; Cat. #S216BA, and #S216BB
Biebrich Scarlet-Acid Fuchsin Solution	Poly Scientific R&D Corp; Cat. #S125
Phosphotungstic/Phosphomolybdic Acid Solution	Thermo Fisher Scientific; Cat. #A248-100 and #A237-100
Aniline Blue	Poly Scientific R&D Corp; Cat. #S116
Glacial Acetic Acid	Thermo Fisher Scientific; Cat. #A491-212

Table S2. List of Machines Used in the Study

Machine	Supplier
Leica TP1020 Automatic Benchtop Tissue Processor	Leica Biosystems
Tissue-Tek® TEC™ 6	Sakura Finetek
HM 325 Microtome	Microm
BZ-X810	Keyence
Mini Gel Tank	Invitrogen
PowerEase™ Touch Power Supply	Invitrogen
iBlot™ 2 Gel Transfer Device	Invitrogen
Azure Sapphire™ Biomolecular Imager	Azure Biosystems
CFX96 C1000 Touch™ Thermal Cycler	Bio-Rad
Sonos 5500 Ultrasound Platform	Hewlett Packard
15 MHz Transducer	Philips

Table S3. List of Antibodies Used in the Study

Antibody	Supplier and Cat. No.
Src-α-actin	Sigma-Aldrich; Cat. #A2172
CD14	Abbiotec; Cat. #251561
iNOS	Abcam; Cat. #ab15323
HMGB1	Abcam; Cat. ab79823
TLR4	Abcam; Cat. #ab13556
NLRP3	Abcam; Cat. #ab214185
Caspase-1	Abcam; Cat. #ab138483
IL-1β	Abcam; Cat. #ab9722
IL-18	Abcam; Cat. #ab71495
GSDMD	Abcam; Cat. #ab219800
CD206	Abcam; Cat. #ab64693
Arginase-1	Santa Cruz; Cat. #sc-18351
IL-10	Abcam; Cat. #ab189392
Alexa Fluor® 568	Invitrogen; Cat. #A11011
iNOS	Abcam; Cat. #ab178945
IL-18	Abcam; Cat. #ab207323
GSDMD	Abcam; Cat. #ab209845
TGF-β1	Abcam; Cat. #ab92486
pSMAD2	Cell Signaling; Cat. #18338
pSMAD3	Cell Signaling; Cat. #9520
GAPDH	Cell Signaling; Cat. #5174
HRP-Linked Antibody	Cell Signaling; Cat. #7074

Table S4. List of Primers Used in the Study

Target	Forward Primer	Reverse Primer
TNF-α	5'-CACACTCAGATCATCTTCCAAAA-3'	5'-GCAATGACTCTAAGTAGACCTGC-3'
IL-6	5'-AGTTGCCTTCTTGGGACTGA-3'	5'-TCCACGATTTCCCAGAGAAC-3'
NLRP3	5'-CACGAGTCCTGGTGACTTTGTA-3'	5'-CAGCCCTTTCGAGGGTCTC-3'
Caspase-1	5'-GAAACGCCATGGCTGACAAG-3'	5'-CGTGCCTTGTCCATAGCAGT-3'
GSDMD	5'-GACTCTGGAGAACTGGTGCC-3'	5'-ACACAGAACTCTGCTCCTGC-3'
IL-1β	5'-AACCTGCTGGTGTGTGACTTC-3'	5'-CAGCACGAGGCTTTTTTGT-3'
IL-18	5'-ACTTTGGCCGACTTCACTGT-3'	5'-GTCTGGTCTGGGGTTCACTG-3'
IL-10	5'-AACCTGCTGGTGTGATTC-3'	5'-CACAGGGGAGAAATCGATACA-3'
GAPDH	5'-ACCCAGAAGACTGTGGATGG-3'	5'-CACATTGGGGGTAGGAACAC-3'
